# Feasibility and acceptability of wearing a neuromodulation device at night in individuals in recovery from opioid use disorder

**DOI:** 10.3389/fpsyt.2024.1481795

**Published:** 2024-11-29

**Authors:** Kristy L. Meads, Steve Huettner, Dexter Amata, Hailey Johnson, Jaime K. Devine, Shenali Warnakulasuriya, Keith R. Murphy, Cameron H. Good

**Affiliations:** ^1^ Attune Neurosciences, Bel Air, MD, United States; ^2^ Stevenson University, Owings Mills, MD, United States; ^3^ Institutes for Behavior Resources, Baltimore, MD, United States

**Keywords:** addiction, sleep, insomnia, human factors, focused ultrasound, sex differences, methadone, ePro

## Abstract

**Introduction:**

Opioid use disorder (OUD) is a serious and persistent problem in the United States with limited non-pharmacological treatment options, especially for the concomitant sleep disorders experienced by most individuals with addiction. While new, non-invasive interventions such as low-intensity focused ultrasound (LIFU) have shown promise in targeting the brain regions impacted throughout addiction and recovery, the devices used are not amenable to outpatient treatment in their current form factor and cannot be used at night during sleep. To bridge this gap and provide a much-needed treatment option for repeated, at-home use, we developed a wearable LIFU device out-of-clinic use.

**Methods:**

This study evaluated the feasibility and acceptability of the portable treatment device among individuals recovering from OUD in an unsupervised, at-home setting. 31 subjects were recruited from a Baltimore, Maryland (USA) outpatient treatment facility and, along with a separate group of 14 healthy controls (HC), were asked to wear a prototype EEG-only (non-LIFU) device for 7 consecutive nights to assess their willingness and adherence to nightly use. Participants used a smartphone application, TrialKit (ePRO), to self-report nightly sleep data (e.g. duration, quality, possible disturbances, and device comfort).

**Results:**

Of the 31 OUD participants recruited, 30 (97%) successfully completed the at-home study, and the majority responded that they would participate in future studies using the head wearable device (OUD, 87%; HC, 71%). OUD participants were statistically more likely than HCs to respond that they would consider using the device in the future to help them sleep (OUD, 70%; HC, 29%). Despite some participants facing technological issues (e.g. lack of reliable phone access or cellular data plans), the OUD group demonstrated high study compliance on par with the healthy control group.

**Discussion:**

Participant’s daily ePRO and exit interview results established that at-home use of advanced treatment technology is feasible in a population group challenged with recovering from OUD. Even more so, numerous participants noted strong willingness to participate in future LIFU-enabled intervention studies to address their persistent sleep issues during recovery.

## 1 Introduction

The Centers for Disease Control and Prevention (CDC) National Center for Health Statistics reports that over 80,000 people died each year from an opioid overdose in 2021, 2022, and 2023 (1). As undergoing opioid agonist treatment (OAT) decreases the risk of overdose for individuals with opioid use disorder (OUD) by nearly 50% (2), encouraging adherence to OUD treatment is imperative in the fight against the opioid epidemic. Unfortunately, relapse is a defining feature of OUD due to the difficulty of opioid withdrawal symptoms (3, 4), and poor sleep health can persist for years following treatment initiation. Not surprisingly, poor sleep is a primary driver of relapse (5–7). Numerous reports confirm that more than 70% of OUD patients undergoing OAT self-report sleep disturbances or disorders (5, 8–10), underscoring its prevalence.

Sleep disturbances persist across the addiction, withdrawal, and recovery process, with inability to sleep serving as a feed-forward stressor that can contribute to relapse. Reports have found inhibition of rapid eye movement (REM) and non-REM (NREM) deep sleep (11–13), decreased total sleep time, and increased wakefulness after sleep onset in those using opioids (14, 15). Longitudinal evaluations indicate that sleep does not naturally improve over the course of OAT with either methadone or buprenorphine use (9, 10, 16). These data suggest that alleviating sleep disturbances in OUD requires targeted and synergistic interventions that are distinct from standard OAT.

Unfortunately, historically prescribed pharmacological interventions for mitigating sleep disturbances can further perpetuate addictive behavior among people recovering from OUD (17–19). The combined use of opioids and sleep aids increases the risk of overdose (20) and non-pharmacological treatments like cognitive behavioral therapy for insomnia (CBTi) may be ineffective as stand-alone treatments for sleep disturbances in OUD, especially for individuals with comorbid pain disorders (21, 22). These findings indicate a need for new non-pharmacological interventions that directly modify sleep physiology with the goal of improving sleep quality, reducing relapse, and promoting abstinence from drug misuse in individuals undergoing outpatient OAT.

To date, non-invasive brain stimulation therapies like transcranial magnetic stimulation (TMS) and transcranial direct current stimulation (tDCS) have been explored (23–25) for their ability to reduce opioid cravings, while auricular vagus nerve stimulation (aVNS) (26) has been utilized to improve opioid withdrawal symptoms. Encouragingly, these neuromodulatory interventions have shown some efficacy in improving recovery outcomes but are not ideal for targeting the deep brain structures necessary to directly augment sleep.

Low-intensity focused ultrasound (LIFU) has emerged as a promising new non-invasive tool for stimulating or suppressing intact brain circuits with great potential for treating a range of psychiatric disorders and sleep issues (27–30). The differentiating feature of LIFU is that ultrasound (US) beams can be focused onto specific deep regions of the brain without impacting surrounding tissue (31–33), unlike with electrical or magnetic stimulation. Unfortunately, until now, the technology has been limited to in-hospital use and, as a result, could not readily translate to routine addiction intervention. To overcome this barrier to treatment, Attune Neurosciences developed a portable, offline MRI-guided head-worn LIFU device for in-clinic or at-home use to manage addiction and treat sleep disturbances. The device is designed to be comfortably worn while real-time electroencephalography (EEG) is analyzed for sleep stage and cortical phase determination, allowing for personalized LIFU stimulation treatment based on a person’s real-time physiological data.

Here, we conducted a 7-night at-home sleep EEG study (*no therapy was administered*) to evaluate nightly device usability and gauge acceptance and adherence of use in the target population, individuals in recovery from OUD. We also examined nightly device usability and adherence in a smaller cohort of healthy controls (HCs) to compare the results and determine if differences exist between the populations. Given the high incidence of sleep disturbances in people with OUD, and the challenge of pharmaceutically treating them, we hypothesized that this group would be amenable to alternative neuromodulation approaches and demonstrate use of the technology on par with the HC group.

## 2 Methods

### 2.1 Study design

This study was designed to evaluate the feasibility and acceptability of nightly wear of a head-worn neuromodulation device among individuals recovering from OUD, as compared to HCs. While no therapy was delivered, participants wore the prototype EEG-only device for 7 consecutive nights in an unsupervised, at-home setting. Nightly sleep EEG data was collected to ensure proper device wear. Survey data was collected daily via a smartphone electronic patient-reported outcomes (ePRO) application (TrialKit; Crucial Data Solutions) to monitor, in real-time, participant perceptions of the device and any issues encountered.

The TrialKit ePRO platform developed by Crucial Data Solutions is purpose-built as a data capture system for clinical trials and medical research. Its functionality extends into electronic health record integration and data collection for regulatory submission. User acceptance testing was performed by Attune prior to study initiation to verify that the application’s functionality met the needs of the study, and captured data accurately for future analysis.

### 2.2 Participant recruitment

All study procedures were approved by Advarra, an independent institutional review board. The addiction arm of the study was conducted in partnership with REACH IBR (Recovery Enhanced by Access to Comprehensive Healthcare, Institute for Behavioral Resources) in Baltimore, Maryland (USA), to recruit and enroll individuals who are in recovery from OUD. The REACH IBR treatment center provides behavioral counseling in addition to daily pharmacological support (e.g. methadone). Study participants were referred for recruitment by councilors or responded to flyers posted throughout the facility. Healthy control participants were recruited via college students through network referral. An index patient was recruited, and subsequent referrals were made via snowball sampling. Participants were compensated $25 per day for their time and effort to wear the device overnight, charge it, and answer daily survey questions. To encourage study adherence and device return, participants received a $100 bonus at their exit interview for wearing the device and completing daily surveys for all 7 nights (34). Visa gift cards were utilized for participant payment, consistent with best practices in populations with addiction (35).

#### 2.2.1 Inclusion and exclusion criteria

Participant recruitment was open to either sex, 18 - 65 years old (inclusive). Criteria included: English speaking, no illegal drug use in the prior 30 days, a working smartphone and data plan for daily survey application use, no use of a continuous positive airway pressure (CPAP) device that could interfere with the head wearable tested in this study, and residing at the same address for the past 30 days (stable sleeping environment). Participants in the addiction arm of the study additionally must have been diagnosed with OUD and be undergoing outpatient OAT with either methadone or buprenorphine. In contrast, HC subjects were free from a substance use diagnosis and were not dependent on other substances except nicotine.

#### 2.2.2 Participant retention

**Figure 1:**
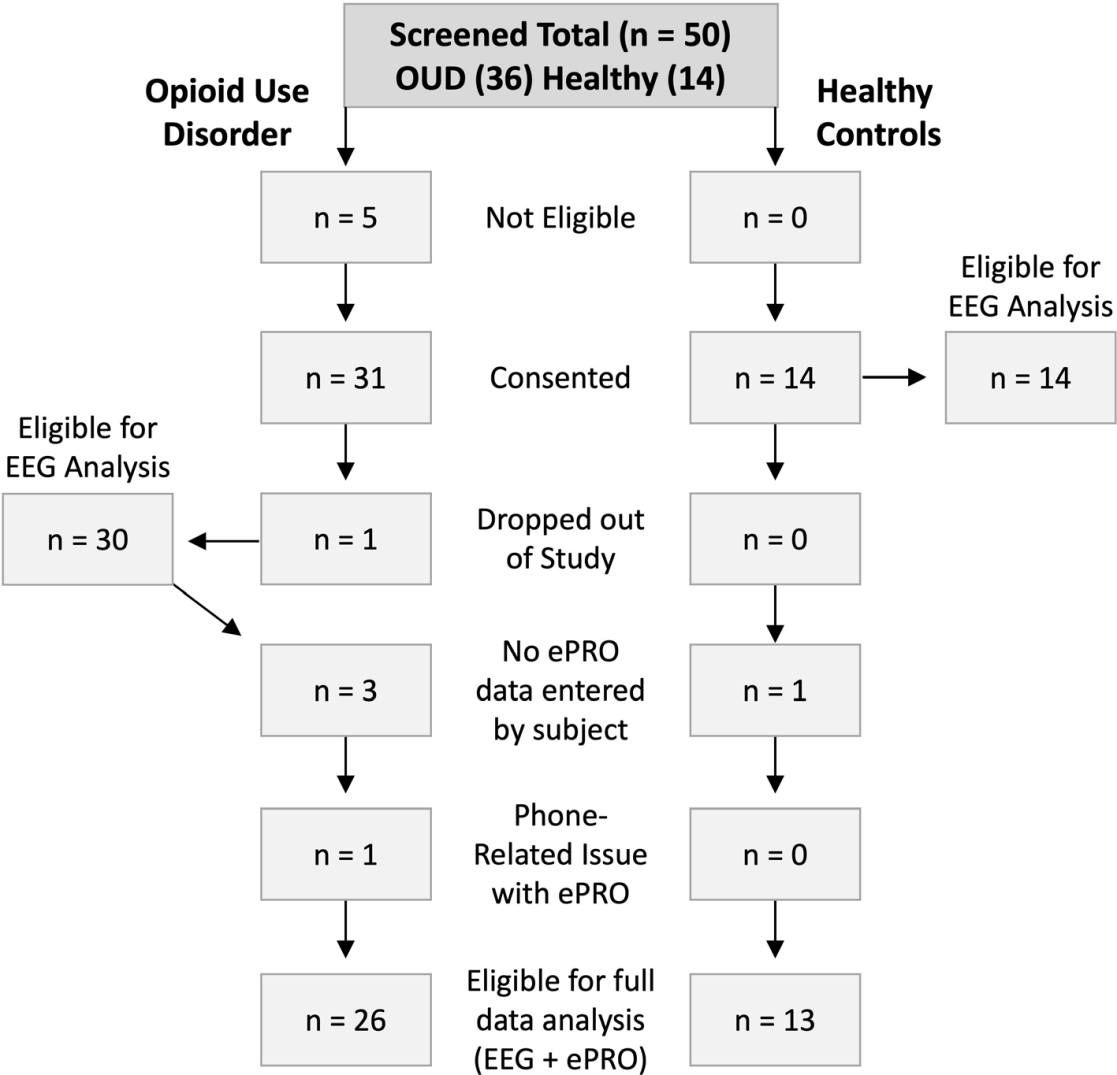
Flow diagram of participant recruitment and retention for both OUD and HC groups.

A flow diagram of participant retention is presented in **Figure 1**. Participants were screened for study inclusion based on the criteria previously described. Five individuals in the OUD group were excluded from further participation due to recent drug use (n = 2) or lack of working smartphone (n = 3). In the OUD and HC groups, 31 (17 females) and 14 (4 females) participants were consented, respectively. A single male participant in the OUD group dropped out due to device discomfort on the first night of wear. During the exit interview, it was determined that the participant was sleeping on a friend’s recliner and should have been excluded from study participation based on lack of stable sleeping environment. Of the 30 remaining OUD participants, 4 either did not answer daily survey questions or had issues with the ePRO application, while 1 HC participant did not answer survey data. Nightly EEG data was available for 30 OUD participants and 14 healthy controls, while 26 and 13 participants also completed the ePRO surveys, respectively.

### 2.3 Study procedure

Participants were electronically screened to determine eligibility. Those eligible for participation were automatically redirected to an electronic consent form. Once consented, participants provided information on age, drug treatment history, traumatic brain injury (TBI) history, skin sensitivities, difficulty sleeping, relationship status, and smoking history. After enrolling participants in the TrialKit ePRO survey platform, the study coordinator assisted them in downloading and logging into the TrialKit phone application and completing a practice questionnaire. Participants were then fitted with the Attune head-wearable prototype device and provided verbal and written instructions for use, as well as a device charger and necessary EEG electrode and device cleaning consumables. Participants were scheduled for an exit interview seven days later to return the device and answer follow-up questions. Each morning upon waking, participants were expected to complete a survey questionnaire via TrialKit and charge their wearable device. Participants were responsible for replacing or re-wetting hydrogel EEG electrode pads nightly before wear. Upon study completion, participants underwent an exit interview to assess overall acceptance, feasibility and willingness of using the device in a future clinical study. All participants were incentivized to complete the week-long study with a prepaid debit card provided at the completion of their exit interview.

#### 2.3.1 Survey collection

During the initial study consent, participants were asked what time to be contacted in the morning regarding daily survey completion. Text messages were sent through the TrailKit phone application reminding participants to answer the daily survey if they had not already done so prior to their agreed upon time. Those who did not complete the survey within an hour were sent one follow-on reminder text by the study coordinator. Complete versions of each survey can be found in the **Supplementary Materials** (**Supplementary Tables S1–S4**).

#### 2.3.2 Head-worn device

The clinical head-worn LIFU device includes bilateral ultrasound transducers that interface with the “temporal window,” a thin portion of skull bone posterior to the eyes that allows access to centralized deep brain structures. A silicone pad overlaid on the US transducer offers contouring and comfort. The front band of the clinical device has integrated 2-channel EEG to measure cortical brain activity, 3-axis accelerometry for capturing head movement, and temperature and photoplethysmography sensors. Custom padding maximizes comfort, and a detachable nose bridge-centered fit-tool allows the user to repeat positioning of the US transducers across use sessions.

**Figure 2:**
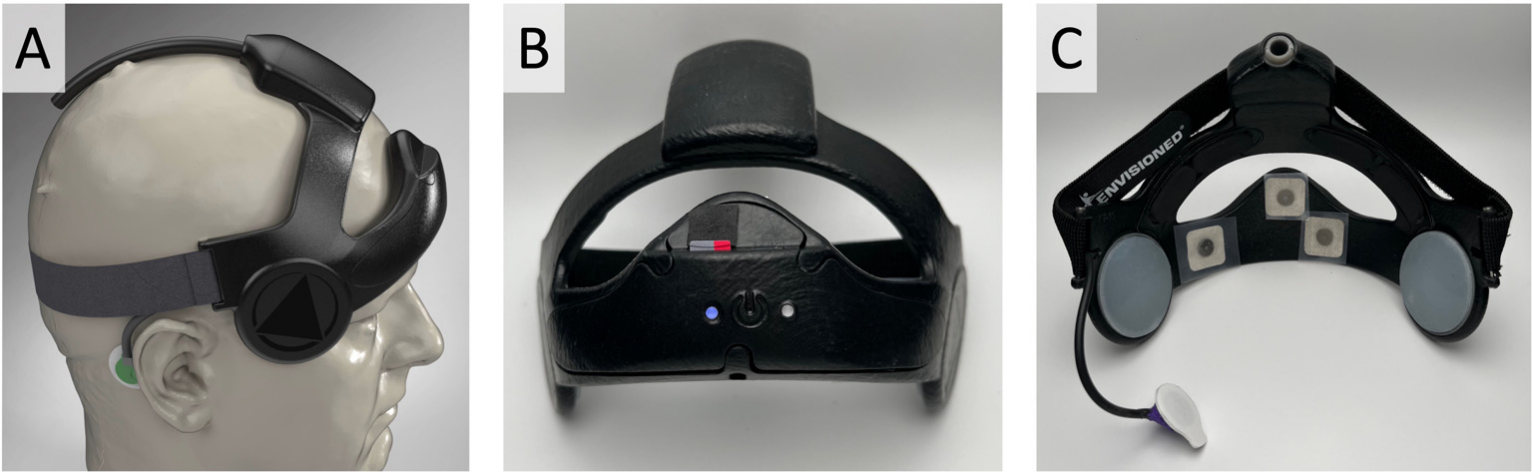
Wearable device worn by participants for each night of the study. **(A)** Side profile 3D rendering of a head model wearing the device. The mastoid EEG reference is shown in green behind the right ear. The circle over the right temple is the ultrasound transducer found in the clinical device. **(B)** Frontal photograph of the wearable human factors device with the SD card inserted, and blue LED power light demonstrating proper device function. **(C)** Rear photograph of the wearable with EEG and mastoid electrodes installed. The rear elastic band is pulled over the top of the device as it is done when donning the device.

Here, we tested a non-clinical human factors version of the device (**Figure 2**) that was geometrically identical to the clinical device but was powered by an integrated rechargeable battery. The device was stripped of all LIFU components (*no therapy delivery*) and replaced with 3D-printed resin surrogates to match the fit and feel of the clinical version. The housing was made from a 3D-printed thermoplastic polyamide elastomer (TPA) material that was free of dyes and secured to the head via an elastic and Velcro rear band. A pair of active EEG sensors (ConMed Softrace Small ECG hydrogel electrodes) were included in the front band approximately at FP1 and FCZ (**Figure 2C**), both referenced to the mastoid (Kendall H124SG ECG electrode), with data streamed and stored at 250 Hz on a local micro-SD memory card for offline analysis. An embedded 3-axis accelerometer (250 Hz) captured head motion throughout use. Applying pressure to a central power button on the front of the device allowed users to turn it on and off.

The human factors device utilized mini-light emitting diodes (LEDs) embedded into the circuit board, and light pipes channeled the diffuse light to the front of the wearable to signal device functionality. When plugged in, an orange or red LED on the right side of the device indicated charging still in progress while a green light indicated charging is complete. The left LED indicator light would glow blue when powered on if the device was adequately charged and a memory card was properly inserted (**Figure 2B**). If the left light was red or orange after powering on, the memory card was likely not inserted correctly or had malfunctioned. Participants were instructed to only use the device if it was properly charged, and the left light indicator was blue during operation.

### 2.4 Data analysis

Statistical analysis was performed using GraphPad Prism Version 10.2.3. Discrete data was summarized using median and median absolute deviation (MAD), while continuous data was summarized using mean and standard deviation (SD). A two-sided p < 0.05 was used to determine statistical significance and adjusted for multiple comparisons, as needed.

#### 2.4.1 Nightly EEG recordings

After each participant completed the study, the micro-SD cards were removed from the wearable devices for analysis. A custom Python script was generated to batch-process each nightly EEG file. The data processing pipeline began with extracting raw EEG and accelerometer signals from the binary data files, followed by the EEG signals being de-medianed to ensure accurate amplitude representation and to remove very low frequency oscillations. The EEG signals then underwent band-power extraction, including Fourier transformation, to assess frequency content over time (10 second bins, 0.1 Hz resolution). Two graphs were generated, one displaying the 3-axis accelerometry and a spectrogram showing the EEG time frequency signal of the entire night recording. The EEG graph and raw data were manually reviewed for regions of electrical noise contamination, which can be seen as high power across multiple frequency bands, or disconnected electrodes which present as horizontal banding in the spectrogram. Regions of data deemed noisy were manually reviewed for confirmation. Each night of EEG recording was then categorized into “5 - Very Good”, “4 - Good”, “3 - Moderate”, “2 - Poor”, and “1 - Very Poor” based on the length and quality of EEG signal (**Supplementary Figure S1**). Recordings considered “Very Good” displayed clean readable data (>80%) and had both EEG channels recording properly without noise contamination or electrode disconnection. Data considered “Good” displayed clean EEG data (70-80%) but may have only one EEG channel with properly recorded data. Data categorized as “Moderate” showed identifiable EEG signal patterns (50-70%) and contained only one properly recorded EEG channel. “Poor” or “Very Poor” data had little to no identifiable EEG signal patterns (<50%) across the two EEG channels. Files with insufficient duration (< 60 minutes) were also classified as “Very Poor”. Due to firmware issues with the EEG acquisition circuit, timestamps were not included with the signal data. This precluded a more in-depth analysis and comparison of sleep/wake cycles between the OUD and HC groups, as originally intended.

#### 2.4.2 TrialKit ePRO daily surveys and exit Interview

Survey data was compiled for each question across all 7 nights of the study and analyzed using descriptive statistics, which was also used to describe demographic and participant histories. Prevalence of smoking and sleep issues were analyzed across study groups and biological sex using a Chi-square (Fisher’s exact) test.

## 3 Results

### 3.1 Demographics

Participant demographics were obtained during consent and are included in **Table 1**. For the OUD group, the mean age was 45.1 ± 13.0 years, while the HCs were younger at 22.6 ± 10.0 years. The OUD group was comprised of 56.7% females (OUD-F), while the HCs were 28.6% female (HC-F). No participants in either group reported skin or scalp sensitivities. A statistically greater proportion of responding OUD participants reported difficulty sleeping (OUD, 67.9%; HC, 21.4%; P = 0.0080), with no difference between males and females in either group (OUD, P = 0.23; HC, P = 0.99; **Supplementary Table S5**). The OUD participants were statistically more likely to smoke cigarettes compared to healthy controls (OUD, 78.6%; HC, 14.3%, P = 0.0001), with no difference between males and females (OUD, P = 0.65; HC, P = 0.066; **Supplementary Table S5**).

**Table 1:** Participant demographics.

	OUD Participants (N = 30)	Healthy Controls (N = 14)
Age		
Mean [SD]	45.1 [13.0]	22.6 [10.0]
Median	42	20
Sex		
Male	13	10
Female	17	4
Marital status		
Single	15	9
In a relationship	5	3
Married	2	0
Living with partner	4	1
Widowed	1	0
Separated	1	0
Divorced	0	1
NR	2	0
Drug treatment		
Yes	24	0
No	4	14
NR	2	0
Number of times in drug treatment		
Average	2.1	–
Median	2	–
Traumatic brain injury		
Yes	1	0
No	19	14
NR	10	0
Skin sensitivity (head or scalp)		
Yes	0	0
No	20	14
NR	10	0
Sleep difficulties		
Yes	19	3
No	9	11
NR	2	0
Nicotine consumption		
Yes	22	2
No	6	12
NR	2	0

### 3.2 ePRO responses

Upon waking each morning, participants were required to login to the ePRO application (TrialKit) and respond to a series of questions regarding their nightly sleep and their experience using the wearable device. Additional questions focused on lifestyle factors that could influence their sleep and perceptions of device use. Nearly all participants in both groups completed the required seven days of ePRO surveys (**Table 2**). There was one HC and four OUD participants who did not use the ePRO system because of technology issues related to phone and text reminders; hence, no self-reported data were available for these participants (**Figure 1**).

**Table 2:** ePRO and EEG data yield.

Participant group		Participants (N)	ePRO responses [median, MAD]	Avg nightly ePRO recorded sleep duration (hrs) [mean, SD]	Avg total nightly EEG recordings (hrs) [mean, SD]	Avg nightly usable EEG recordings (hrs) [mean, SD]	Avg nightly non-usable EEG recordings (hrs) [mean, SD]
OUD	Male	13	7, 0	8.14, 1.81	10.26, 5.41	5.62, 2.61	4.64, 4.02
	Female	17	6, 1	8.05, 1.33	11.33, 6.50	5.26, 3.76	6.07, 4.81
Healthy Controls	Male	10	7, 0	7.34, 0.91	12.34, 4.36	5.66, 3.24	6.68, 4.99
	Female	4	7, 1	8.16, 2.08	15.40, 4.52	7.78, 1.29	7.62, 5.63

#### 3.2.1 Sleep

From the available ePRO data, participants across groups and biological sexes self-reported similar nightly total sleep time (TST) durations (ANOVA, P = 0.28) that were greater than the associated TST duration of acquired EEG data (mean difference ± sd; OUD-M, 2.96 ± 3.74 hrs; OUD-F, 2.18 ± 3.12 hrs; HC-M, 1.71 ± 3.12 hrs; HC-F, 0.38 ± 1.98 hrs). Nightly EEG data were analyzed for signal quality and continuity of recording throughout each night to confirm device wear by the participants. Most of the device-recorded EEG data from both participant groups received a qualitative score of 3 or higher (Moderate quality; **Supplementary Figure S1**) (OUD-M, 59.4%; OUD-F, 52.5%; HC-M, 61.0%; HC-F, 60.7%).

From the available ePRO data, participants reported similar median sleep quality ratings (Median, IQR [25^th^, 75^th^ Percentile]; OUD-M, 3 [2.5, 4]; OUD-F, 3 [2, 4]; HC-M, 3 [3, 4]; HC-F, 4 [3, 4]; **Table 3**), with the only statistical difference being between OUD-M and HC-M (Kruskal-Wallis, P = 0.024). In addition to self-reported sleep ratings, participants also commented on the disruption of sleep, and other possible sleep factors such as the wearable’s blue LED power light, exercise, caffeine, and/or alcohol use within four hours of bedtime. Regarding the blue light, the majority of participant’s nightly responses were reported as either “No” or “N/A” on the disruption of their personal (OUD-M, 88%; OUD-F, 79%; HC-M, 91%; HC-F, 88%) or their partner’s (OUD-M, 91%; OUD-F, 79%; HC-M, 90%; HC-F, 91%; **Table 3**) sleep. A majority of responses in both groups also indicated “No” to exercising (OUD-M, 96%; OUD-F, 98%; HC-M, 86%; HC-F, 92%; **Table 4**) and caffeine/energy drink use within four hours of wearing the device and going to sleep (OUD-M, 85%; OUD-F, 96%; HC-M, 89%; HC-F, 83%; **Table 4**).

**Table 3:** Participant sleep quality rating and reported nighty sleep disruption from the blue power LED indicator.

Participant group		N	Median sleep rating - 99% CI (upper, lower)	Did the blue light disrupt your sleep last night?			Did the blue light disrupt the sleep of anyone who slept by you last night?			
				Yes	No	N/R	Yes	No	N/A	N/R
OUD	Male	13	3 (3, 3)	3 (4%)	64 (88%)	6 (8%)	1 (1%)	59 (81%)	7 (10%)	6 (8%)
	Female	17	3 (3, 4)	3 (3%)	73 (79%)	16 (17%)	4 (4%)	64 (70%)	8 (9%)	16 (17%)
Healthy Controls	Male	10	3 (3, 4)	1 (2%)	51 (91%)	4 (7%)	2 (4%)	20 (36%)	30 (54%)	4 (7%)
	Female	4	4 (3, 4)	1 (4%)	21 (88%)	2 (8%)	0 (0%)	14 (58%)	8 (33%)	2 (8%)

**Table 4:** Reported nightly alcohol and caffeine use and exercise within 4 hours of going to sleep.

Participant group		N	Did you drink alcohol yesterday within 4 hrs of going to sleep?			Did you consume any caffeine or energy drinks yesterday within 4 hrs of going to sleep?			Did you exercise yesterday within 4 hrs of going to sleep?		
			Yes	No	N/R	Yes	No	N/R	Yes	No	N/R
OUD	Male	13	6 (8%)	67 (92%)	0 (0%)	11 (15%)	62 (85%)	0 (0%)	3 (4%)	70 (96%)	0 (0%)
	Female	17	1 (1%)	89 (97%)	2 (2%)	3 (3%)	88 (96%)	1 (1%)	2 (2%)	90 (98%)	0 (0%)
Healthy Controls	Male	10	20 (36%)	36 (64%)	0 (0%)	6 (11%)	50 (89%)	0 (0%)	8 (14%)	48 (86%)	0 (0%)
	Female	4	10 (42%)	13 (54%)	1 (4%)	4 (17%)	20 (83%)	0 (0%)	2 (8%)	22 (92%)	0 (0%)

Concerning alcohol use between the broader groups, the HC participants (which mostly consisted of college students) were statistically more likely to drink alcohol within four hours of going to sleep than the OUD participants (Fisher’s Exact Test, P < 0.0001). Within the OUD group, OUD-M participants were statistically more likely to drink alcohol as compared to OUD-F (Fisher’s Exact Test, P = 0.046); no statistical difference was found between HC-M and HC-F (Fisher’s Exact Test, P = 0.612). When we examined nightly responses to alcohol use, OUD participants consistently responded with “No” to alcohol use within four hours of going to sleep (OUD-M, 92%; OUD-F, 97%). This is in contrast to the HC participants who were less likely to respond with “No” to nightly alcohol use (HC-M, 64%; HC-F, 54%; **Table 4**).

#### 3.2.2 Medication use

Based on ePRO self-reported data on nightly medication use (**Table 5**), a higher percentage of females with OUD took prescribed medication at least once during the study (OUD-M, 8%; OUD-F, 41%), although this was not statistically different from males (P = 0.09). Similarly, there was no statistical difference in unprescribed (over-the-counter, OTC) medication use before bed between males and females with OUD (P = 0.11). Examples of reported prescribed medications include Ambien, Clonidine, Gabapentin, Strattera, Propanol, Prazosin, and Seroquil; while reported OTC medications included Ibuprofen, Tylenol, Benadryl, Advil, and Allegra. In contrast, none of the HC participants (HC-M, 0%; HC-F, 0%) reported taking any prescribed medications. In both groups, only the females reported taking OTC medications (OUD-F, 24%; HC-F, 25%). Across the total study nights, OUD-F were statistically more likely to take prescribed (Fischer’s Exact, P < 0.0001) and OTC (Fischer’s Exact, P = 0.035) medications, as compared to OUD-M (**Table 5**).

**Table 5:** Reported medication use across participant groups.

Participant group		Participants (N) [# of ePRO responses]	Prescribed medication		Unprescribed (OTC) medication	
			# of participants who took at least 1 prescribed medication	Total # of nights where participants took prescribed medication before going to sleep	# of participants who took at least 1 unprescribed medication	Total # of nights where participants took unprescribed medication before going to sleep
OUD	Male	13 [73]	1 (8%)	1 (1%)	0 (0%)	0 (0%)
	Female	17 [92]	7 (41%)	29 (32%)	4 (24%)	6 (7%)
Healthy Controls	Male	10 [56]	0 (0%)	0 (0%)	0 (0%)	0 (0%)
	Female	4 [24]	0 (0%)	0 (0%)	1 (25%)	1 (2%)

#### 3.2.3 Wearable device comfort

**Figure 3:**
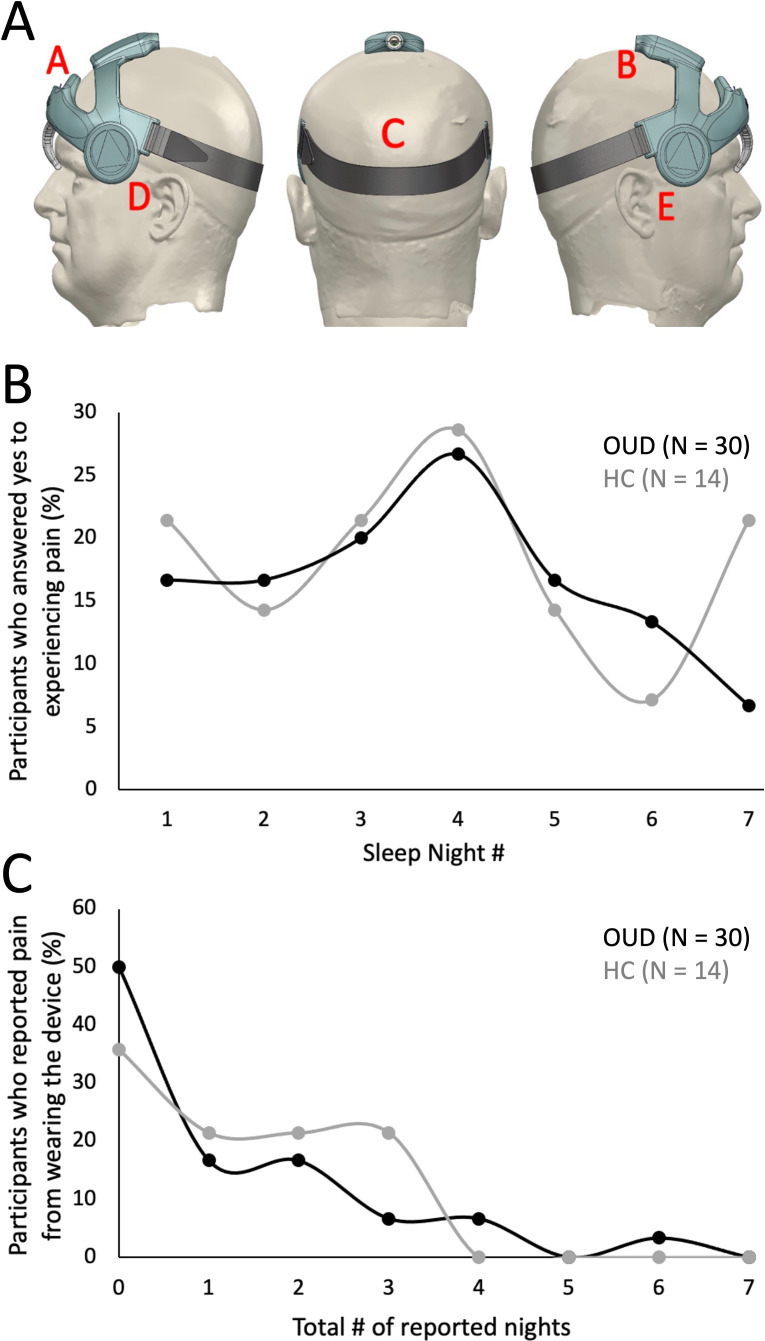
Assessment of device comfort. **(A)** Illustration depicting possible areas of discomfort as shown in the participant’s daily ePRO surveys (A: Forehead, B: Top of head, C: Back of head, D: Left side of ear, E: Right side of ear). **(B)** Percentage of participants who answered “Yes” to experiencing discomfort across each study night. **(C)** Percentage of participants who reported experiencing device discomfort. Each participant’s daily response (No, 0; Yes, 1) was summed to determine the cumulative number of nights where they reported discomfort.

**Figure 3A** was used to determine potential areas of discomfort caused by the head wearable device during sleep. Based on the total nightly reports of any pain occurrence (**Table 6**), most responses across all groups reported “0 or (No Pain)” from the wearable device (OUD-M, 79%; OUD-F, 73%; HC-M, 84%; HC-F, 58%). Only a few responses indicated participants experiencing pain at multiple (at least 3) different locations while wearing the device (OUD-M, 1%; OUD-F, 4%; HC-M, 0%; HC-F, 8%). From the total nightly pain location reports (**Table 7**), there was no statistical difference between OUD-M and OUD-F participants who reported pain across the different regions of the head (P_A_ = 0.14; P_B_ = 0.13; P_C_ > 0.99; P_D_ = 0.55; P_E_ = 0.79). This was similar in the HC group except at the forehead location, where females were statistically more likely to report discomfort (P_A_ = 0.0025) compared to other locations (P_B_ > 0.99; P_C_ > 0.99; P_D_ = 0.17; P_E_ = 0.06).

**Table 6:** Occurrence of reported head pain across number of locations and participant groups.

Participant group		Participants (N) [# of ePRO responses]	# of nights where pain was reported at (X) number of location(s)					
			0 (No Pain)	1	2	3	4	5
OUD	Male	13 [73]	58 (79%)	12 (16%)	1 (1%)	0 (0%)	0 (0%)	1 (1%)
	Female	17 [92]	67 (73%)	8 (9%)	9 (10%)	4 (4%)	0 (0%)	0 (0%)
Healthy Controls	Male	10 [56]	47 (84%)	5 (9%)	4 (7%)	0 (0%)	0 (0%)	0 (0%)
	Female	4 [24]	14 (58%)	2 (8%)	5 (21%)	2 (8%)	0 (0%)	0 (0%)

**Table 7:** Reported number of instances of pain at each identified location on the head.

Pain Location			A	B	C	D	E
Participant group		Participants (N) [# of ePRO responses]	Forehead	Top of head	Back of head	center side of ear	Right side of ear
OUD	Male	13 [73]	5 (7%)	1 (1%)	1 (1%)	4 (5%)	8 (11%)
	Female	17 [92]	14 (15%)	6 (7%)	2 (2%)	8 (9%)	8 (9%)
Healthy Controls	Male	10 [56]	1 (2%)	0 (0%)	2 (4%)	6 (11%)	4 (7%)
	Female	4 [24]	6 (25%)	0 (0%)	0 (0%)	6 (25%)	6 (25%)

The total nightly percentage of OUD and HC participants who reported any head pain from the wearable is shown in **Figure 3B**. The highest percentage of participants who reported pain occurred at night four (OUD, 27%; HC, 29%). There was an initial increase in the percentage of OUD participants who reported pain from night one through four (17% - 27%), then a steady decrease through night seven (27% - 7%). In contrast, there was no generalizable trend of reported pain for the HC group. **Figure 3C** shows that most participants did not experience discomfort across multiple nights of device use. For instance, in the OUD group only 7% of participants reported pain for four, 0% for five, 3% for six and 0% for seven nights of wearing the device; while, no HC participant (0%) reported experiencing discomfort from the device on more than four nights of the study (**Figure 3C**).

### 3.3 Exit interviews

After completing the seven-night study, participants answered exit interviews where they reported on their overall experience with the wearable device, ePRO system, and other aspects of the study. From the verbal exit interview, nearly all participants reported that the device was “Easy” or “Very Easy” to don at night (OUD-M, 100%; OUD-F, 100%; HC-M, 100%; HC-F, 75%; **Supplementary Table S6**); there was a single female participant who reported having a “Very Difficult” time donning the device. Similarly, nearly all participants also reported that nightly replacement of the EEG electrodes was “Easy” or “Very Easy” (OUD-M, 100%; OUD-F, 94%; HC-M, 90%; HC-F, 100%; **Supplementary Table S6**). The remaining two participants (one from each group) reported having “Moderate” difficulty in the nightly task. Open ended responses to the verbal exit interviews were categorized based on response characteristics. When asked about challenges with the ePRO system, participants in both groups experienced various technical issues (OUD, 40%; HC, 29%; Fisher’s Exact Test, P = 0.52) with using the application (**Supplementary Table S6**). These issues varied from complete inability to respond daily, initial setup challenges with cellular data plans and lack of phone memory storage, or challenges after night one that required study coordinator intervention.

In terms of device comfort, participants reported it as being “Moderate” to “Very Comfortable” to wear (OUD-M, 70%; OUD-F, 95%; HC-M, 70%; HC-F, 50%; **Supplementary Table S6**). From the Likert responses, all participants in both groups (100%) either “Agree” or “Strongly Agree” that overall, the device was easy to use (**Supplementary Table S7**) and the majority of participants would participate in future studies using the head wearable device (OUD, 87%; HC, 71%). When participants were asked if they would consider using the device in the future to help them sleep, the OUD group was statistically more likely to respond positively than the HC group (OUD, 70%; HC, 29%; Fisher’s Exact Test, P = 0.02; **Supplementary Table S7**).

### 3.4 User feedback

During the exit interview, we inquired about overall device comfort and usability (**Supplementary Table S3**). Notable feedback focused on the participants desire to improve sleep without the use of pharmacology. For instance, one OUD participant who has been drug-free for 10 months, the longest continuous period in 30 years, stated that his only lingering issue is his inability to sleep. He described how this negatively impacts his employment and creates issues with his marriage. This individual was very enthusiastic about new technology and responded that “If I could use [the] device for sleep, without using drugs, Hell yah!”. Similarly, another OUD participant responded that the device “was comfortable to wear at night and liked the idea of not having to take medications [to improve sleep]”. Another participant noted that the device “was comfortable, and was able to sleep with it” and it was “something you could get used to wearing every night”, while another participant noted “I didn’t even know I was wearing it”. Two female OUD participants reported “sleeping better with the device” due to the “cooling effect of the silicone pads” on their temples. Similarly in the HC group, participants noted the device “was comfortable, didn’t realize I was wearing it” and “comfort was good, forehead felt fine”.

Participants who slept on their side and/or moved a lot during sleep reported mild discomfort. For instance, participants “felt a slight pressure” and “side discomfort” when sleeping on their side. As a result, the same participant “slept fine only on some nights” and “other nights I had to adjust it”, while another participant “felt more comfortable sleeping on [their] back” despite normally being a side sleeper. Regarding hair types, participants with longer and smoother hair had issues keeping the elastic rear band in place and had to “adjust the band several time at night”. Nightly wear also presented a challenge for a participant with dreadlocks.

Critical responses around the study requirements focused on use and connectivity issues associated with the TrialKit (ePRO) application. It took some participants a few days to become familiar with the interface, as several had “problems with app”, “was confused about pin # [number]”, “issue with time stamp(ing) on [the] first two days”, and “kept hitting save but wouldn’t go through”. While others felt the interface was easy to use and had no issues with daily login and reporting. In terms of connectivity, several participants noted temporarily losing internet access for a day and as result failed to complete the previous night’s report. Similarly, OUD participants who had limited cellular data plans were unable to receive daily SMS text reminders and failed to complete their daily report.

## 4 Discussion

This project represents the first step towards developing a functional, non-pharmacological therapeutic device specifically designed to target sleep issues in an OUD population. The current study focused on the comfort of participants while wearing the device, a key component to treatment success that is frequently underappreciated; comfort is integral to treatment adherence and routine use. CPAP machines serve as an example of an effective, non-pharmacological treatment for disordered sleep (obstructive sleep apnea; OSA) in which treatment success suffers due to patient non-adherence. Issues related to patients’ discomfort are consistently found to predict non-adherence (36), while improving patient comfort has been found to increase adherence rates in CPAP users (37). In 2023, ResMed, a pioneer in the CPAP field, spent approximately $288 million on its research and development efforts (38). The newly released AirFit F40 CPAP mask highlights the company’s focus on creating ultra compact, adjustable, and comfortable devices that fit a broad population range and is critical to improving compliance. Drawing from CPAP user experience, it is imperative to study the degree of comfort that target populations have when wearing a treatment device, which can determine future adherence and therapeutic success.

Here, we tested device comfort and usability in a target population of individuals in recovery from OUD. Results demonstrated a near perfect adherence rate to wearing the device for seven consecutive nights (median = 7 nights), and a high completion rate where 30/31 (97%) consented participants finished the full study. This suggests that the head wearable device was comfortable to sleep with and any discomfort experienced was not sufficient to discontinue nightly use, or warranted dropping out of the study. We also had 100% of the test devices returned during the exit interview and in fully operational condition. This is key to future clinical implementation in a home setting, as it suggests that the participants followed device use and daily care instructions provided to them at the onset of the study.

Our results parallel and extend a pilot study of 8 male participants who underwent nightly EEG monitoring during supervised opioid withdrawal in a residential unit. The participants wore a forehead mounted, battery operated wireless EEG device (Sleep Profiler; Advanced Brain Monitoring, Carlsbad, CA) for 85.6% of the scheduled nights, but compliance varied based on the stage of withdrawal (39). Another study used the same EEG device during a 7 night at-home assessment of sleep in individuals with OUD, with an observed 75% compliance across a larger cohort of 55 participants (15). Our device compliance was higher than both studies, possibly due to our participant section of only individuals who have been free from drug use for over 30 days and having a stable home sleeping environment. Like our results, these studies observed discrepancies between subjective sleep diaries and objective EEG data such that sleep diaries tended to over-estimate TST versus objective EEG measures. However, our results should be interpreted cautiously, as our numbers are particularly discrepant and likely influenced by issues with the EEG recording hardware that precluded a more in-depth signal processing analysis (see Limitations).

From the daily ePRO responses, females reported more OTC and prescribed medication use than males. This is in line with prior research indicating that females are more likely to take medication for sleep, prescribed or OTC, than males (40). Females are also more likely to report pain and are known to have more pain sensitivity (41) that can lead to higher rates of medication use. Females are statistically more likely to develop conditions resulting in excessive pain, such as osteoarthritis, inflammatory arthropathy, and fibromyalgia (42) that can lead to chronic use of pain medications. This mirrors what we found in this study, with females using Tylenol, Advil, Ibuprofen, while males did not report use of any of these OTC medications. Severe pain conditions can result in the need to treat pain with opioids, which females are 1.5 times more likely to fill prescriptions for than males (43), which use can lead to addiction.

The study design required participants to use a cell phone-based application (TrialKit) to answer daily survey questions about device comfort. This approach has been successfully used in prior studies by us, and others, with similar subject populations (44). Further, prior research has shown that individuals who have a history of drug injection have high cell phone usage and are comfortable using technology (45). Here, following consent, the research coordinator assisted participants in loading TrialKit onto their phones, which allowed them to answer questions each morning upon awakening (46). Data was used to track, in real-time, their perceptions of the wearable device and any issues encountered. Study participants unanimously expressed a willingness to use TrialKit, but it did pose a challenge for a minority of individuals, particularly those reliant on government sponsored phones and data plans that have limited storage space that restricts application download options. The research coordinator, on many occasions, worked with study participants to remove unwanted or unused applications to free up storage space to install TrialKit (47). This should be taken into consideration in future research that involves smartphone-based technology and applicants should be screened accordingly.

During the exit interview, we asked participants if they would like to be contacted about future clinical research studies that involve delivery of LIFU to potentially help them sleep. The OUD participants were statistically more likely to positively respond versus HC participants (OUD, 70%; HC 29%), which is in line with the percent of each population that reported trouble sleeping during initial study consent (OUD, 68%; HC, 21%). These numbers also approximate published numbers on sleep disorders in individuals with OUD and who are undergoing OAT (70%) (5, 8–10). Encouragingly, it demonstrates the desire of these individuals to embrace non-pharmacological treatment options. As technology advances, options to treat sleep disorders and other components of addiction will continue to grow.

LIFU represents one such technology and which underlies the clinical version of the wearable device tested here. In contrast to other approaches, LIFU allows precise neuromodulation of centralized deep brain structures such as the thalamus (48), hypothalamus, amygdala (49), nucleus accumbens (NAc) (50), and hippocampus. These, and additional brain regions, serve as tractable targets for addressing components of addiction, the sleep/wake system, and for treating psychiatric disorders (51), demonstrating the highly versatile nature of the technology. Indeed, recent published evidence suggests that targeting LIFU to the NAc, a critical core region of addiction, can dramatically reduce cue-induced drug craving for a range of substances that includes cocaine, alcohol, nicotine, and opioids (52, 53). Remarkably, these results persist for many weeks post-treatment, suggesting that LIFU could be a promising new therapeutic approach with outcomes that rival that of pharmaceuticals while avoiding side effects. While encouraging, the technology used in those studies is limited to in-hospital use and does not readily translate to routine addiction treatment. Further advances in wearable devices that broaden treatment options to community addiction centers such as REACH IBR, and at-home use, offer tremendous potential for addressing the persistent opioid epidemic.

### 4.1 Next steps

Based on current results of intermittent EEG connectivity in some participants, we aim to improve conformal fit of the device by reducing the form-factor of our current EEG components and transitioning to a flexible circuit design with interchangeable front band. A follow-on study utilizing the current design could be performed to evaluate engineering improvements on nightly data collection for statistical comparison to the current dataset. Due to the demonstrated success of working directly with an OUD population for feasibility and acceptability testing, future clinical trials will focus on treating multiple aspects of addiction (e.g., drug craving and underlying sleep disorders) to reduce rates of relapse. The ATTN201 is particularly amenable to this, as it can be used in an at-home, out-of-clinic, setting in contrast to the LIFU system used in prior addiction treatment studies that requires an active MRI for neuronavigation (52, 53).

### 4.2 Limitations

Study limitations include only testing one device form factor; thus, it is unknown how these results translate to other head wearable devices. We experienced firmware issues with EEG record timing and a sensitive power button precluded a comparative analysis of EEG and sleep features between OUD and HC participants. In TrialKit, participants should not have been allowed to leave daily survey questions unanswered; thus, resulting in non-responses (NR) to some questions. This feature was tested prior to implementation, and it remains unknown how this materialized during study use. OUD participants were recruited from a single recovery clinic in Baltimore, MD (USA), although our participant sample included a broad range of demographics and number of times in drug treatment.

To simulate real-world device usage, participants were allowed to continue their medications, including sleep aids, and consume alcohol, which may have impacted overnight perceptions of wearing the device. There was a notable age discrepancy between the OUD and HC groups, where the mean age of the OUD group was >20 years older than the HC, which could influence their perception and usage of the device. Future clinical studies using LIFU therapy will more tightly age match across groups. Participants were compensated for nightly device wear and daily survey responses, and provided a bonus for completing all 7 nights and returning the device upon study conclusion. This payment schedule was based on our study coordinators history in working with similar populations with addiction in Baltimore, MD, and common practices with this population (34, 35).

## 5 Conclusions

This study demonstrated that Attune’s head-worn medical device is feasible for at-home, nightly use among individuals with OUD, opening the door to future addiction treatment options with LIFU. Strong study adherence (e.g., daily survey completion, device wear and maintenance, and return of the research device to the study coordinator) also indicates that with proper study design and support, the recovering OUD population can effectively participate in longer-duration, outpatient studies.

## Data Availability

The original contributions presented in the study are included in the article/**Supplementary Material**. Further inquiries can be directed to the corresponding author.

## References

[1] NCDAS. Opioid Crisis Statistics [2023]: Prescription Opiod Abuse (2024). Available online at: https://drugabusestatistics.org/opioid-epidemic/ (accessed August 5, 2024).

[2] Santo T Clark B Hickman M Grebely J Campbell G Sordo L. Association of opioid agonist treatment with all-cause mortality and specific causes of death among people with opioid dependence: A systematic review and meta-analysis. JAMA Psychiatry. (2021) 78:979–93. doi: 10.1001/jamapsychiatry.2021.0976 PMC8173472 34076676

[3] Clark RE Baxter JD Aweh G O’Connell E Fisher WH Barton BA. Risk factors for relapse and higher costs among medicaid members with opioid dependence or abuse: opioid agonists, comorbidities, and treatment history. J Subst Abuse Treat. (2015) 57:75–80. doi: 10.1016/j.jsat.2015.05.001 25997674 PMC4560989

[4] Dunn KE Weerts EM Huhn AS Schroeder JR Andrew TD Bigelow GE. Preliminary evidence of different and clinically-meaningful opioid withdrawal phenotypes. Addict Biol. (2020) 25:e12680. doi: 10.1111/adb.12680 30295400 PMC6546557

[5] Burke CK Peirce JM Kidorf MS Neubauer D Punjabi NM Stoller KB. Sleep problems reported by patients entering opioid agonist treatment. J Subst Abuse Treat. (2008) 35:328–33. doi: 10.1016/j.jsat.2007.10.003 PMC2626437 18248944

[6] Dijkstra BAG De Jong CAJ Krabbe PFM van der Staak CPF. Prediction of abstinence in opioid-dependent patients. J Addict Med. (2008) 2:194–201. doi: 10.1097/ADM.0b013e31818a6596 21768990

[7] Lydon-Staley DM Cleveland HH Huhn AS Cleveland MJ Harris J Stankoski D. Daily sleep quality affects drug craving, partially through indirect associations with positive affect, in patients in treatment for nonmedical use of prescription drugs. Addict Behav. (2017) 65:275–82. doi: 10.1016/j.addbeh.2016.08.026 PMC5140682 27544697

[8] Stein MD Herman DS Bishop S Lassor JA Weinstock M Anthony J. Sleep disturbances among methadone maintained patients. J Subst Abuse Treat. (2004) 26:175–80. doi: 10.1016/S0740-5472(03)00191-0 15063910

[9] Dunn KE Finan PH Andrew Tompkins D Strain EC. Frequency and correlates of sleep disturbance in methadone and buprenorphine-maintained patients. Addict Behav. (2018) 76:8–14. doi: 10.1016/j.addbeh.2017.07.016 28735039 PMC5614840

[10] Peles E Schreiber S Hetzroni T Adelson M Defrin R. The differential effect of methadone dose and of chronic pain on pain perception of former heroin addicts receiving methadone maintenance treatment. J Pain. (2011) 12:41–50. doi: 10.1016/j.jpain.2010.04.009 20561825

[11] Dimsdale JE Norman D DeJardin D Wallace MS. The effect of opioids on sleep architecture. J Clin Sleep Med. (2007) 3:33–6. 17557450

[12] Cronin A Keifer JC Baghdoyan HA Lydic R. Opioid inhibition of rapid eye movement sleep by a specific mu receptor agonist. Br J Anaesth. (1995) 74:188–92. doi: 10.1093/bja/74.2.188 7696070

[13] Cheatle MD Webster LR. Opioid therapy and sleep disorders: risks and mitigation strategies. Pain Med. (2015) 16:S22–6. doi: 10.1111/pme.12910 PMC4608386 26461072

[14] Sharkey KM Kurth ME Anderson BJ Corso RP Millman RP Stein MD. Assessing sleep in opioid dependence: a comparison of subjective ratings, sleep diaries, and home polysomnography in methadone maintenance patients. Drug Alcohol Depend. (2011) 113:245–8. doi: 10.1016/j.drugalcdep.2010.08.007 PMC3025068 20850231

[15] Finan PH Mun CJ Epstein DH Kowalczyk WJ Phillips KA Agage D. Multimodal assessment of sleep in men and women during treatment for opioid use disorder. Drug Alcohol Depend. (2020) 207:107698. doi: 10.1016/j.drugalcdep.2019.107698 31816489 PMC9351606

[16] Nordmann S Lions C Vilotitch A Michel L Mora M Spire B. A prospective, longitudinal study of sleep disturbance and comorbidity in opiate dependence (the ANRS Methaville study). Psychopharmacol (Berl). (2016) 233:1203–13. doi: 10.1007/s00213-016-4202-4 26753792

[17] Rush CR Baker RW. Differential effects of zolpidem and triazolam on a digit-enter-and-recall task with varying delay intervals. Behav Pharmacol. (1999) 10:S78. doi: 10.1097/00008877-199908001-00200

[18] Daugherty JL Hendricks L Simpson C. Sleep aids: sedative-hypnotic drugs in America. National Forum Journal of Counseling and Addiction. (2024) 3(1):1–5.

[19] Fathi HR Yoonessi A Khatibi A Rezaeitalab F Rezaei-Ardani A. Crosstalk between sleep disturbance and opioid use disorder: A narrative review. Addict Health. (2020) 12:140–58. doi: 10.22122/ahj.v12i2.249 PMC7395935 32782736

[20] Szmulewicz A Bateman BT Levin R Huybrechts KF. The risk of overdose with concomitant use of Z-drugs and prescription opioids: A population-based cohort study. Am J Psychiatry. (2021) 178:643–50. doi: 10.1176/appi.ajp.2020.20071038 33900810

[21] Jones JD Mogali S Comer SD. Polydrug abuse: A review of opioid and benzodiazepine combination use. Drug Alcohol Depend. (2012) 125:8–18. doi: 10.1016/j.drugalcdep.2012.07.004 22857878 PMC3454351

[22] McCrae CS Curtis AF Miller MB Nair N Rathinakumar H Davenport M. Effect of cognitive behavioural therapy on sleep and opioid medication use in adults with fibromyalgia and insomnia. J Sleep Res. (2020) 29:e13020. doi: 10.1111/jsr.13020 32126156 PMC7483285

[23] Shen Y Cao X Tan T Shan C Wang Y Pan J. 10-hz repetitive transcranial magnetic stimulation of the left dorsolateral prefrontal cortex reduces heroin cue craving in long-term addicts. Biol Psychiatry. (2016) 80:e13–4. doi: 10.1016/j.biopsych.2016.02.006 26995024

[24] Wang Y Shen Y Cao X Shan C Pan J He H. Transcranial direct current stimulation of the frontal-parietal-temporal area attenuates cue-induced craving for heroin. J Psychiatr Res. (2016) 79:1–3. doi: 10.1016/j.jpsychires.2016.04.001 27115508

[25] Taremian F Nazari S Moradveisi L Moloodi R. Transcranial direct current stimulation on opium craving, depression, and anxiety: A preliminary study. J ECT. (2019) 35:201. doi: 10.1097/YCT.0000000000000568 30664050

[26] Miranda A Taca A. Neuromodulation with percutaneous electrical nerve field stimulation is associated with reduction in signs and symptoms of opioid withdrawal: a multisite, retrospective assessment. Am J Drug Alcohol Abuse. (2018) 44:56–63. doi: 10.1080/00952990.2017.1295459 28301217

[27] Tyler WJ Lani SW Hwang GM. Ultrasonic modulation of neural circuit activity. Curr Opin Neurobiol. (2018) 50:222–31. doi: 10.1016/j.conb.2018.04.011 29674264

[28] Bystritsky A Korb AS. A review of low-intensity transcranial focused ultrasound for clinical applications. Curr Behav Neurosci Rep. (2015) 2:60–6. doi: 10.1007/s40473-015-0039-0

[29] Tyler WJ Tufail Y Finsterwald M Tauchmann ML Olson EJ Majestic C. Remote excitation of neuronal circuits using low-intensity, low-frequency ultrasound. PloS One. (2008) 3. doi: 10.1371/journal.pone.0003511 PMC2568804 18958151

[30] Yaakub SN White TA Roberts J Martin E Verhagen L Stagg CJ. Transcranial focused ultrasound-mediated neurochemical and functional connectivity changes in deep cortical regions in humans. Nat Commun. (2023) 14:5318. doi: 10.1038/s41467-023-40998-0 37658076 PMC10474159

[31] Legon W Sato TF Opitz A Mueller J Barbour A Williams A. Transcranial focused ultrasound modulates the activity of primary somatosensory cortex in humans. Supplementary information. Nat Neurosci. (2014) 17:322–9. doi: 10.1038/nn.3620 24413698

[32] Legon W Ai L Bansal P Mueller JK. Neuromodulation with single-element transcranial focused ultrasound in human thalamus. Hum Brain Mapping. (2018) 39:1995–2006. doi: 10.1002/hbm.23981 PMC6866487 29380485

[33] Mueller J Legon W Opitz A Sato TF Tyler WJ. Transcranial focused ultrasound modulates intrinsic and evoked EEG dynamics. Brain Stimul. (2014) 7:900–8. doi: 10.1016/j.brs.2014.08.008 25265863

[34] Dominguez D Jawara M Martino N Sinaii N Grady C. Commonly performed procedures in clinical research: A benchmark for payment. Contemp Clin Trials. (2012) 33:860–8. doi: 10.1016/j.cct.2012.05.001 PMC3408804 22580210

[35] Anderson E McNair L. Ethical issues in research involving participants with opioid use disorder. Ther Innov Regul Sci. (2018) 52:280–4. doi: 10.1177/2168479018771682 PMC5944085 29714588

[36] Mehrtash M Bakker JP Ayas N. Predictors of continuous positive airway pressure adherence in patients with obstructive sleep apnea. Lung. (2019) 197:115–21. doi: 10.1007/s00408-018-00193-1 30617618

[37] Catcheside PG. Predictors of continuous positive airway pressure adherence. F1000 Med Rep. (2010) 2:70. doi: 10.3410/M2-70 20948830 PMC2954420

[38] ResMed Inc. Announces Results for the Fourth Quarter of Fiscal Year 2023. Resmed Inc (2024). Available at: https://newsroom.resmed.com/news-releases/news-details/2023/ResMed-Inc.-Announces-Results-for-the-Fourth-Quarter-of-Fiscal-Year-2023/default.aspx.

[39] Dunn KE Finan PH Huhn AS Gamaldo C Bergeria CL Strain EC. Wireless electroencephalography (EEG) to monitor sleep among patients being withdrawn from opioids: Evidence of feasibility and utility. Exp Clin Psychopharmacol. (2022) 30:1016–23. doi: 10.1037/pha0000483 PMC8648854 34096756

[40] Reuben C. Sleep medication use in adults aged 18 and over: United States, 2020. NCHS Data Brief. (2023) 462. doi: 10.15620/cdc:123013 36700855

[41] Bartley EJ Fillingim RB. Sex differences in pain: a brief review of clinical and experimental findings. Br J Anaesth. (2013) 111:52–8. doi: 10.1093/bja/aet127 PMC3690315 23794645

[42] Templeton KJ. Sex and gender issues in pain management. J Bone Joint Surge. (2020) 102:32–5. doi: 10.2106/JBJS.20.00237 32251123

[43] Schieber LZ Guy GP Seth P Losby JL. Variation in adult outpatient opioid prescription dispensing by age and sex — United states, 2008–2018. MMWR Morb Mortal Wkly Rep. (2020) 69:298–302. doi: 10.15585/mmwr.mm6911a5 32191686 PMC7739983

[44] Swendsen J. Contributions of mobile technologies to addiction research. Dialogues Clin Neurosci. (2016) 18:213–21. doi: 10.31887/DCNS.2016.18.2/jswendsen PMC4969708 27489461

[45] Hudgens S Kern S Barsdorf AI Cassells S Rowe A King-Kallimanis BL. Best practice recommendations for electronic patient-reported outcome dataset structure and standardization to support drug development. Value Health. (2023) 26:1242–8. doi: 10.1016/j.jval.2023.02.011 36849080

[46] Antoine D Heffernan S Chaudhry A King V Strain EC. Age and gender considerations for technology-assisted delivery of therapy for substance use disorder treatment: A patient survey of access to electronic devices. Addictive Disord Their Treat. (2016) 15:149–56. doi: 10.1097/ADT.0000000000000088 PMC5425165 28503100

[47] Ozga JE Paquette C Syvertsen JL Pollini RA. Mobile phone and internet use among people who inject drugs: Implications for mobile health interventions. Subst Abus. (2022) 43:592–7. doi: 10.1080/08897077.2021.1975871 PMC9536021 34491889

[48] Cain JA Spivak NM Coetzee JP Crone JS Johnson MA Lutkenhoff ES. Ultrasonic thalamic stimulation in chronic disorders of consciousness. Brain Stimul. (2021) 14:301–3. doi: 10.1016/j.brs.2021.01.008 33465497

[49] Chou T Deckersbach T Guerin B Sretavan Wong K Borron BM Kanabar A. Transcranial focused ultrasound of the amygdala modulates fear network activation and connectivity. Brain Stimul. (2024) 17:312–20. doi: 10.1016/j.brs.2024.03.004 38447773

[50] Peng X Connolly DJ Sutton F Robinson J Baker-Vogel B Short EB. Non-invasive suppression of the human nucleus accumbens (NAc) with transcranial focused ultrasound (tFUS) modulates the reward network: a pilot study. Front Hum Neurosci. (2024) 18:1359396. doi: 10.3389/fnhum.2024.1359396 38628972 PMC11018963

[51] Fan JM Woodworth K Murphy KR Hinkley L Cohen JL Yoshimura J. Thalamic transcranial ultrasound stimulation in treatment resistant depression. Brain Stimul. (2024) 17:1001–4. doi: 10.1016/j.brs.2024.08.006 PMC11531731 39173737

[52] Mahoney JJ Haut MW Carpenter J Ranjan M Thompson-Lake DGY Marton JL. Low-intensity focused ultrasound targeting the nucleus accumbens as a potential treatment for substance use disorder: safety and feasibility clinical trial. Front Psychiatry. (2023) 14:1211566. doi: 10.3389/fpsyt.2023.1211566 37779628 PMC10540197

[53] Mahoney JJ Thompson-Lake DGY Ranjan M Marton JL Carpenter JS Zheng W. Low-intensity focused ultrasound targeting the bilateral nucleus accumbens as a potential treatment for substance use disorder: A first-in-human report. Biol Psychiatry. (2023) 94:e41–3. doi: 10.1016/j.biopsych.2023.06.031 37610405

