# Supplementary material for: Feasibility and acceptability of wearing a neuromodulation device at night in individuals in recovery from opioid use disorder
Source: Front Psychiatry. 2024 Nov 29;15:1481795. doi: 10.3389/fpsyt.2024.1481795 (PMC11640868; doi:10.3389/fpsyt.2024.1481795)
Supplement: Supplementary file 1 [file SupplementaryFile1.docx]

Supplementary Material

Feasibility and acceptability of wearing a neuromodulation device at night in individuals in recovery from opioid use disorder

Kristy L Meads^1^, Steve Huettner^1^, Dexter Amata^1^, Hailey Johnson^1,2^, Jaime Devine^3^, Shenali Warnakulasuriya^1^, Keith R Murphy^1^, Cameron H Good^1^*

^1^ Attune Neurosciences, Bel Air, Maryland, USA

^2^ Stevenson University, Owings Mills, Maryland, USA

^3^ Institute for Behavioral Research, Baltimore, Maryland, USA

***Correspondence:** Cameron H. Good, Ph.D.; cgood@attuneneuro.com

## Supplementary Figure 1: Qualitative classification of processed 2-channel EEG data collected nightly by the wearable. A single triaxial accelerometer embedded in the device captured head movement. EEG data was manually scored by two researchers and rated based on data quality. Ratings ranged from 5 (best) to 1 (worst) based on the amount of noise present in each EEG channel. The horizontal banding shown in the examples below are caused by EEG electrode disconnect from the user’s forehead.


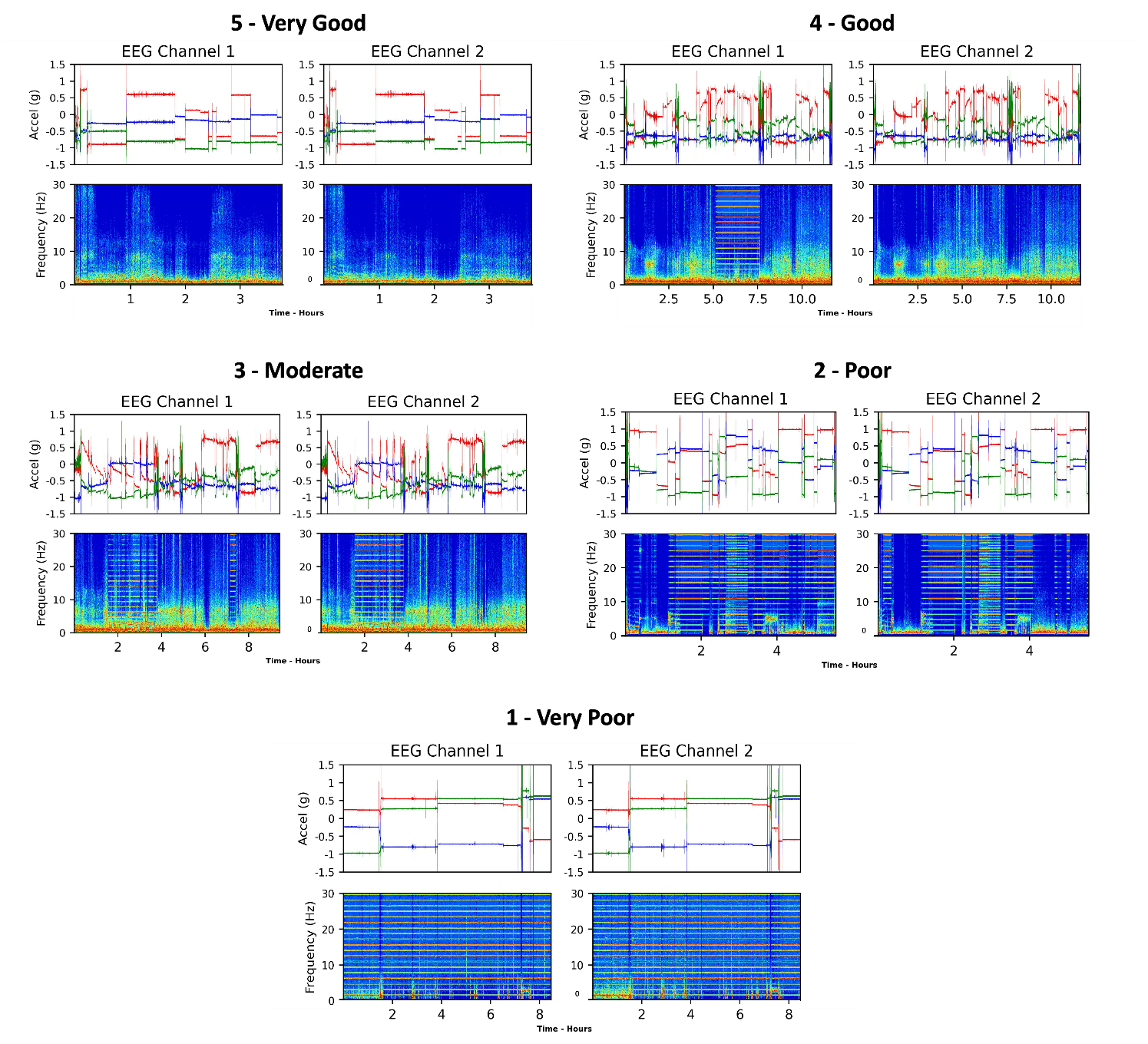


## Supplementary Table 1: Consent questionnaire

| **Question** | **Possible Answer Choices** |
| --- | --- |
| How old are you? | User Response |
| Biological Sex | Male, Female |
| How many times have you been in drug treatment? | User Response |
| How many times have you been in drug treatment? | User Response |
| Have you ever experienced TBI? | Yes, No |
| Do you have skin sensitivities on your head or scalp? | Yes, No |
| Do you have difficulty failing or staying asleep at night? | Yes, No |
| Which of the following describes your current relationship status? | Single never married, In a Relationship, Living with Partner, Married, Widowed, or Separated |
| Do you Smoke cigarettes? | Yes, No |

## Supplementary Table 2: ePro sleep questionnaire

| **Decode ID** | **Question** |
| --- | --- |
| S1 | Did you wear the Attune headband while sleeping last night? |
| S2 | What time did you go to sleep last night? |
| S3 | What time did you wake up? |
| S4 | How would you rate your sleep? |
| S5 | Did you drink alcohol yesterday within 4 hours of going to sleep? |
| S6 | Did you consume any caffeine or energy drinks yesterday within 4 hours of going to sleep? (Ex. Coffee, Redbull, Monster etc.) |
| S7 | Did you have nicotine yesterday within 4 hours of going to sleep? (Ex. Smoking cigarettes, vaping, tobacco etc.) |
| S8 | Did you take any prescribed medications yesterday within 4hrs of going to sleep? |
| S9 | Did you take any unprescribed medication yesterday within 4hrs of going to sleep? |
| S10 | Did you exercise yesterday within 4hrs of going to sleep? |
| S11 | How tired were you when you went to bed? Please rate on a scale of 1 to 5. |
| S12 | Was your sleep last night disrupted or were you woken up by an external factor? (Ex pet, child, storm, phone, bathroom) |
| S12.1 | Approximately how many times was your sleep disrupted? |
| S13 | What was your sleep position for most of the night? |
| S14 | Which type of electrode do you have? |
| S14.1 | What day of electrode wear is it? |
| S15 | Did you replace any of the electrodes on the headband last night? |
| S15.1 | Which electrode(s) did you replace last night? (Drag and drop all that apply) |
| S16 | Was the blue light lit on the headband when you woke up? |
| S16.1.1 | Did the blue light disrupt your sleep last night? |
| S16.1.2 | Did the blue light disrupt the sleep of anyone who slept by you last night? |
| S16.2 | Did you wear the Attune headband the entire time you slept? |
| S16.3 | Did the device hurt your head at any point in the night? |
| Form 185 | Where did it hurt? |

## Supplementary Table 3: Verbal exit interview questions

| **Question** | **Possible Answer Choices** |
| --- | --- |
| How difficult was it to put on the device? | 1-5: 1= Not Difficult, 5= Very Difficult |
| How would you rate the comfort of the device? | 1-5: 1 = Very comfortable (Did not feel the device at all while sleeping), 6= Very uncomfortable (device was unable to be worn while sleeping) |
| How difficult was it to replace the electrodes? | 1-5:1 = Not Difficult, 5 = Very Difficult |
| What was the most difficult part replacing the electrodes? | User Response |
| Were there any issues with the cabling? | Yes, No |
| Were there any issues with the control box | Yes, No |
| What was the biggest challenge (if any) of using the device? | User Response |
| What was the biggest challenge (if any) of using the ePro? | User Response |
| What was the best part of the device? (if any)- Ex, Comfort? Fit? Etc. | User Response |
| Are there any other environmental or device issues or factors you would like to report? | User Response |

## Supplementary Table 4: Likert Exit Interview Questions

| **Question** | **Possible Answer Choices** |
| --- | --- |
| The headset is easy to use. | 1–5: 1=Strongly Disagree, 5=Strongly Agree |
| The headset is something I would consider wearing in the future to help me sleep. | 1–5: 1=Strongly Disagree, 5=Strongly Agree |
| Wearing the headset was comfortable. | 1–5: 1=Strongly Disagree, 5=Strongly Agree |
| Charging the headset every night was difficult. | 1–5: 1=Strongly Disagree, 5=Strongly Agree |
| The blue light affected my sleep. | 1–5: 1=Strongly Disagree, 5=Strongly Agree |
| The blue light affected the sleep of my partner. | 1–5: 1=Strongly Disagree, 5=Strongly Agree or N/A = Not Applicable |
| Attaching the electrode behind my ear was difficult. | 1–5: 1=Strongly Disagree, 5=Strongly Agree |
| Other people who have sleep issues would benefit from this headset. | 1–5: 1=Strongly Disagree, 5=Strongly Agree |
| I would be concerned about how the headset would affect my sleep. | 1–5: 1=Strongly Disagree, 5=Strongly Agree |
| I would like to be contacted about future studies concerning the headset. | 1–5: 1=Strongly Disagree, 5=Strongly Agree |

## Supplementary Table 5: Demographics of sleep difficulties and smoking prevalence

| **Participant group** | | **Participants**  **(N)** | **Sleep difficulties** | | | **Smoke cigarettes** | | |
| --- | --- | --- | --- | --- | --- | --- | --- | --- |
|  |  |  | Yes | No | NR | Yes | No | NR |
| OUD | Male | 13 | 7 (54%) | 6 (46%) | 0 (0%) | 11 (85%) | 2 (15%) | 0 (0%) |
|  | Female | 17 | 12 (71%) | 3 (18%) | 2 (12%) | 11 (65%) | 4 (24%) | 2 (12%) |
| Healthy  Controls | Male | 10 | 2 (20%) | 8 (80%) | 0 (0%) | 0 (0%) | 10 (100%) | 0 (0%) |
|  | Female | 4 | 1 (25%) | 3 (75%) | 0 (0%) | 2 (50%) | 2 (50%) | 0 (0%) |

## Supplementary Table 6: Verbal exit interview across participant groups

|  | **OUD Participants (N = 30)** | | **Healthy Controls (N = 14)** | |  |
| --- | --- | --- | --- | --- | --- |
|  | **Male (N = 13)** | **Female (N = 17)** | **Male (N = 10)** | **Female (N = 4)** |  |
| ***1. How difficult was it to put on the device?*** | | | | | |
| Very Easy | 12 (92%) | 16 (94%) | 8 (80%) | 3 (75%) |  |
| Easy | 1 (8%) | 1 (6%) | 2 (20%) | 0 (0%) |  |
| Moderate | 0 (0%) | 0 (0%) | 0 (0%) | 0 (0%) |  |
| Difficult | 0 (0%) | 0 (0%) | 0 (0%) | 0 (0%) |  |
| Very Difficult | 0 (0%) | 0 (0%) | 0 (0%) | 1 (25%) |  |
| ***2. How would you rate the comfort of the device?*** | | | | | |
| Very Comfortable | 4 (31%) | 3 (18%) | 1 (10%) | 0 (0%) |  |
| Comfortable | 4 (31%) | 3 (18%) | 4 (40%) | 1 (25%) |  |
| Moderate | 1 (8%) | 10 (59%) | 3 (30%) | 1 (25%) |  |
| Uncomfortable | 3 (23%) | 1 (6%) | 2 (20%) | 1 (25%) |  |
| Very Uncomfortable | 1 (8%) | 0 (0%) | 0 (0%) | 1 (25%) |  |
| ***3. How difficult was it to replace the electrodes?*** | | | | | |
| Very Easy | 13 (100%) | 15 (88%) | 8 (80%) | 4 (100%) |  |
| Easy | 0 (0%) | 1 (6%) | 1 (10%) | 0 (0%) |  |
| Moderate | 0 (0%) | 1 (6%) | 1 (10%) | 0 (0%) |  |
| Difficult | 0 (0%) | 0 (0%) | 0 (0%) | 0 (0%) |  |
| Very Difficult | 0 (0%) | 0 (0%) | 0 (0%) | 0 (0%) |  |
| ***7. What was the biggest challenge (if any) of using the device?*** | | | | | |
| None | 10 (77%) | 12 (71%) | 5 (50%) | 0 (0%) |  |
| Sleep Orientation | 2 (15%) | 0 (0%) | 1 (10%) | 0 (0%) |  |
| Firmware Issue | 0 (0%) | 1 (6%) | 0 (0%) | 1 (25%) |  |
| Pain | 0 (0%) | 1 (6%) | 1 (10%) | 2 (50%) |  |
| Other Device Issues | 1 (8%) | 3 (18%) | 3 (30%) | 1 (25%) |  |
| ***8. What was the biggest challenge (if any) of using the ePRO?*** | | | | | |
| No Issues | 8 (62%) | 10 (59%) | 8 (80%) | 2 (50%) |  |
| Day 1 Only Issues | 3 (23%) | 1 (6%) | 0 (0%) | 1 (25%) |  |
| Complicated | 1 (8%) | 2 (12%) | 0 (0%) | 0 (0%) |  |
| Technology Issues | 1 (8%) | 4 (24%) | 2 (20%) | 1 (25%) |  |
| ***9. What was the best part of the device? (If any)- Ex, Comfort? Fit? etc.*** | | | | | |
| None | 3 (23%) | 2 (12%) | 7 (70%) | 1 (25%) |  |
| Comfort | 10 (77%) | 6 (35%) | 2 (20%) | 2 (50%) |  |
| Silicone Pads | 0 (0%) | 4 (24%) | 0 (0%) | 0 (0%) |  |
| Easy to use | 0 (0%) | 2 (12%) | 1 (10%) | 0 (0%) |  |
| Overall Design | 0 (0%) | 4 (24%) | 0 (0%) | 1 (25%) |  |

## Supplementary Table 7: Likert exit interview across participant groups

|  | **OUD Participants (N = 30)** | | **Healthy Controls (N = 14)** | |
| --- | --- | --- | --- | --- |
|  | **Male (N = 13)** | **Female (N = 17)** | **Male (N = 10)** | **Female (N = 4)** |
| ***The headset is easy to use.*** | | | | |
| Strongly Disagree | 0 (0%) | 0 (0%) | 0 (0%) | 0 (0%) |
| Disagree | 0 (0%) | 0 (0%) | 0 (0%) | 0 (0%) |
| Undecided | 0 (0%) | 0 (0%) | 0 (0%) | 0 (0%) |
| Agree | 4 (31%) | 5 (29%) | 3 (30%) | 0 (0%) |
| Strongly Agree | 9 (69%) | 12 (71%) | 7 (70%) | 4 (100%) |
| ***The headset is something I would consider wearing in the future to help me sleep.*** | | | | |
| Strongly Disagree | 1 (8%) | 0 (0%) | 3 (30%) | 0 (0%) |
| Disagree | 1 (8%) | 2 (12%) | 0 (0%) | 1 (25%) |
| Undecided | 2 (15%) | 3 (18%) | 5 (50%) | 1 (25%) |
| Agree | 3 (23%) | 5 (29%) | 2 (20%) | 2 (50%) |
| Strongly Agree | 6 (46%) | 7 (41%) | 0 (0%) | 0 (0%) |
| ***Wearing the headset was comfortable.*** | | | | |
| Strongly Disagree | 0 (0%) | 0 (0%) | 0 (0%) | 0 (0%) |
| Disagree | 3 (23%) | 3 (18%) | 4 (40%) | 2 (50%) |
| Undecided | 2 (15%) | 1 (6%) | 3 (30%) | 0 (0%) |
| Agree | 3 (23%) | 9 (53%) | 2 (20%) | 1 (25%) |
| Strongly Agree | 2 (15%) | 4 (24%) | 1 (10%) | 1 (25%) |
| No Response | 3 (23%) | 0 (0%) | 0 (0%) | 0 (0%) |
| ***Charging the headset every night was difficult.*** | | | | |
| Strongly Disagree | 6 (46%) | 12 (71%) | 8 (80%) | 3 (75%) |
| Disagree | 4 (31%) | 5 (29%) | 2 (20%) | 1 (25%) |
| Undecided | 0 (0%) | 0 (0%) | 0 (0%) | 0 (0%) |
| Agree | 0 (0%) | 0 (0%) | 0 (0%) | 0 (0%) |
| Strongly Agree | 0 (0%) | 0 (0%) | 0 (0%) | 0 (0%) |
| No Response | 3 (23%) | 0 (0%) | 0 (0%) | 0 (0%) |
| ***Attaching the electrode behind my ear was difficult.*** | | | | |
| Strongly Disagree | 5 (38%) | 10 (59%) | 7 (70%) | 4 (100%) |
| Disagree | 5 (38%) | 7 (41%) | 3 (30%) | 0 (0%) |
| Undecided | 0 (0%) | 0 (0%) | 0 (0%) | 0 (0%) |
| Agree | 0 (0%) | 0 (0%) | 0 (0%) | 0 (0%) |
| Strongly Agree | 0 (0%) | 0 (0%) | 0 (0%) | 0 (0%) |
| No Response | 3 (23%) | 0 (0%) | 0 (0%) | 0 (0%) |
| ***I would like to be contacted about future studies concerning the headset.*** | | | | |
| Strongly Disagree | 0 (0%) | 0 (0%) | 1 (10%) | 0 (0%) |
| Disagree | 0 (0%) | 0 (0%) | 0 (0%) | 1 (25%) |
| Undecided | 1 (8%) | 0 (0%) | 2 (20%) | 0 (0%) |
| Agree | 1 (8%) | 2 (12%) | 2 (20%) | 0 (0%) |
| Strongly Agree | 8 (62%) | 15 (88%) | 5 (50%) | 3 (75%) |
| No Response | 3 (23%) | 0 (0%) | 0 (0%) | 0 (0%) |
